# The Clinical Utility of DCISionRT^®^ on Radiation Therapy Decision Making in Patients with Ductal Carcinoma In Situ Following Breast-Conserving Surgery

**DOI:** 10.1245/s10434-021-09903-1

**Published:** 2021-04-05

**Authors:** Chirag Shah, Troy Bremer, Charles Cox, Pat Whitworth, Rakesh Patel, Anushka Patel, Eric Brown, Linsey Gold, David Rock, Lee Riley, Christy Kesslering, Sheree Brown, Robert Gabordi, James Pellicane, Rachel Rabinovich, Sadia Khan, Sandra Templeton, Lonika Majithia, Shawna C. Willey, Fredrik Wärnberg, Naamit K. Gerber, Steve Shivers, Frank A. Vicini

**Affiliations:** 1 Department of Radiation Oncology, Taussig Cancer Institute, Cleveland Clinic, Cleveland, OH USA; 2 PreludeDx, Laguna Hills, CA USA; 3 University of South Florida, Tampa, FL USA; 4 Nashville Breast Center, Nashville, TN USA; 5 Sutter Health, Castro Valley, CA USA; 6 Arizona Center for Cancer Care, Phoenix, AZ USA; 7 Comprehensive Breast Care, Michigan Healthcare Professionals, Troy, MI USA; 8 GenesisCare, Fort Myers, FL USA; 9 St. Luke’s Hospital, Allentown, PA USA; 10 Radiation Oncology Department, Northwestern Medicine, Warrenville, IL USA; 11 Wellstar Radiation Oncology, Hiram, GA USA; 12 BayCare Medical Group, Tampa, FL USA; 13 Bon Secours, Richmond, VA USA; 14 Department of Radiation Oncology, University of Colorado School of Medicine, Aurora, CO USA; 15 Hoag Breast Center, Irvine, CA USA; 16 Department of Surgery, Houston Methodist, Sugar Land, TX USA; 17 Radiation Oncology Associates, Fairfax, VA USA; 18 Schar Cancer Institute, Inova, Fairfax, VA USA; 19 Department of Surgery, Sahlgrenska University Hospital, Gothenburg, Sweden; 20 Department of Radiation Oncology, Laura and Isaac Perlmutter Cancer Center, New York, NY USA; 21 GenesisCare, Michigan Healthcare Professionals, Farmington Hills, MI USA

## Abstract

**Background:**

The role of radiation therapy (RT) following breast-conserving surgery (BCS) in ductal carcinoma in situ (DCIS) remains controversial. Trials have not identified a low-risk cohort, based on clinicopathologic features, who do not benefit from RT. A biosignature (DCISionRT^®^) that evaluates recurrence risk has been developed and validated. We evaluated the impact of DCISionRT on clinicians’ recommendations for adjuvant RT.

**Methods:**

The PREDICT study is a prospective, multi-institutional, observational registry in which patients underwent DCISionRT testing. The primary endpoint was to identify the percentage of patients where testing led to a change in RT recommendations.

**Results:**

Overall, 539 women were included in this study. Pre DCISionRT testing, RT was recommended to 69% of patients; however, post-testing, a change in the RT recommendation was made for 42% of patients compared with the pre-testing recommendation; the percentage of women who were recommended RT decreased by 20%. For women initially recommended not to receive an RT pre-test, 35% had their recommendation changed to add RT following testing, while post-test, 46% of patients had their recommendation changed to omit RT after an initial recommendation for RT. When considered in conjunction with other clinicopathologic factors, the elevated DCISionRT score risk group (DS > 3) had the strongest association with an RT recommendation (odds ratio 43.4) compared with age, grade, size, margin status, and other factors.

**Conclusions:**

DCISionRT provided information that significantly changed the recommendations to add or omit RT. Compared with traditional clinicopathologic features used to determine recommendations for or against RT, the factor most strongly associated with RT recommendations was the DCISionRT result, with other factors of importance being patient preference, tumor size, and grade.

**Supplementary Information:**

The online version contains supplementary material available at 10.1245/s10434-021-09903-1.

The role of adjuvant radiation therapy (RT) following breast-conserving surgery (BCS) for ductal carcinoma in situ (DCIS) has evolved but remains controversial. Initial trials evaluating the role of RT consistently demonstrated a reduction in local recurrence with no survival advantage, which has been confirmed by meta-analyses.^1^–^4^ Subsequent studies that incorporated endocrine therapy continued to demonstrate a benefit for adjuvant RT.^5^ Recently, a second generation of de-intensification trials evaluated RT omission following BCS in low-risk patients (clinical and pathologic factors: grade, margin, size).^6^–^9^ Despite the enrollment of ‘low-risk’ patients, 10-year total local recurrence rates of 10–15% (4–7.5% invasive recurrences) were seen, with no groups of patients identified who did not benefit substantially.^6^–^8^ While no survival advantage has been seen with RT, the goal of DCIS treatment is to prevent invasive breast cancer recurrences (approximately 50% of all subsequent breast events) that are associated with an increased risk of breast cancer mortality.^1^–^5^ Additionally, local recurrence entails further treatment that potentially includes more surgery, radiation, and systemic therapy. That said, RT is not without normal tissue toxicity, financial toxicity, and psychological impact. Therefore, the benefit of RT in reducing local recurrences in DCIS must be balanced against its potential adverse effects.^10^

Since traditional clinical and pathologic features have failed to identify a low-risk cohort of DCIS patients that does not benefit from RT following BCS, there is a need for biologic signatures to assess recurrence risk. One such risk profile, which integrates key cancer biologic pathways and clinicopathologic factors (DCISionRT; PreludeDx, Inc., Laguna Hills, CA, USA), has previously been shown to prognosticate 10-year total and invasive breast cancer risks and also predict the benefit of adjuvant RT.^11^,^12^ As data on this risk profile grows, a key question is how the DCISionRT test affects clinical decision making. The purpose of the PREDICT study was to evaluate the impact of DCISionRT testing on clinicians’ recommendations in routine clinical practice to administer or omit RT in patients with DCIS following BCS.

## Methods

The PREDICT study is an observational, prospective, multicenter study; centers included academic and community practices and the primary investigator at each institution could be surgeons or radiation oncologists. The study was designed to assess the clinical utility of DCISionRT and its impact on decision making in treatment management. Patients were enrolled between February 2018 and July 2020 at 44 centers before DCISionRT testing (electronic supplementary Table 1). Patients included women aged 25 years or older who were treated with BCS for unilateral DCIS, who were eligible to receive adjuvant RT, and had DCISionRT testing as part of routine clinical practice. The protocol allowed for all patients meeting the eligibility criteria to be enrolled. Patients not offered the registry were not tracked. The presence of lobular carcinoma in situ or benign breast disease in addition to DCIS was allowed. Patients were required to enroll in the study within 120 days of diagnosis. Exclusion criteria included a history of prior breast malignancy, evidence of invasive breast cancer (microinvasion, lymph node involvement, or Paget’s disease of the nipple), pregnancy, mastectomy, serious comorbid disease, insufficient tumor tissue, or missing data for the test. The study was approved by the Western Institutional Review Board (WIRB) and by Institutional Review Boards from the 23 participating centers; all participants signed informed consent. The study protocol was registered in the ClinicalTrials.gov database (NCT03448926).

**Table 1:** Patient and disease characteristics

Clinical factor	Radiation oncologist (independently)		Surgeon (independently)		All radiation oncologists (independently or with Tumor Board)		Total	
	*n*	%	*n*	%	*n*	%	*n*	%
*Age, years*								
< 50	26	14	26	11	56	18	82	15
≥ 50	165	86	206	89	250	82	457	85
< 70	144	75	170	73	231	75	402	75
≥ 70	47	25	62	27	75	25	137	25
*Grade*								
1 or 2	129	68	162	70	208	68	371	69
3	62	32	70	30	98	32	168	31
*Size, cm*								
≤ 1	131	69	159	69	199	65	359	66
> 1 and ≤ 2.5	45	24	52	22	76	25	128	24
> 2.5	15	8	21	9	31	10	52	10
*Necrosis*								
Present	90	47	120	52	143	47	263	49
Absent	59	31	71	31	91	30	163	30
Unknown	42	22	41	17	72	23	113	21
*Margin*								
Positive	3	2	2	1	7	2	9	2
Close	27	14	41	18	42	14	83	15
Negative	161	84	189	81	257	84	447	83
*Race*								
African American	22	12	25	11	35	11	61	11
Caucasian	157	82	191	82	249	81	440	82
Other	12	6	16	7	22	7	38	7
*Ethnicity*								
Hispanic	10	5	14	6	10	3	24	4
Non-Hispanic	176	92	214	92	289	95	504	94
Unknown	5	3	4	2	7	2	11	2
*RTOG 9804*								
Good risk	95	50	113	49	154	50	268	50
Not good risk	96	50	119	51	152	50	271	50
*ECOG E5194*								
Grade 1 or 2 low risk	102	53	116	50	163	53	280	52
Grade 3 low risk	36	19	37	16	48	16	85	16
Not low risk	53	28	79	34	95	31	174	32

Total patient count includes surgeon (*n* = 232), radiation oncology (*n* = 306), and medical oncology (*n* = 1) physician specialties

*RTOG* Radiation Therapy Oncology Group, *ECOG* Eastern Cooperative Oncology Group

The DCISionRT test was developed specifically for women with DCIS to provide an individualized 10-year risk profile (total and invasive breast events) after treatment with BCS ± RT, with confidence intervals provided. The DCISionRT is performed using formalin-fixed, paraffin-embedded tumor tissue. The test is a biologic signature that uses a panel of seven protein biomarkers (COX-2, FOXA1, HER2, Ki-67, p16/INK4A, PgR, and SIAH2), based on work from the University of California, San Francisco, to assess key pathways in the tumor biology in combination with four clinicopathologic factors, including margin status, which together provide an individualized risk assessment. The test uses a non-linear algorithm that relies on the interactions of the biomarkers and clinicopathologic factors to generate a DCISionRT test result, including the continuous decision score (DS) with associated risk, as well as categorical DS Low (DS ≤ 3) and DS Elevated (DS > 3) risk groups.

Patient demographics and clinicopathologic characteristics were recorded. A preliminary recommendation for adjuvant RT was made by the treating clinician based on the clinicopathologic features, and reported along with patient preference before and after test results. As this was a registry rather than a randomized trial, no standard criteria for offering RT were provided. Rather, the registry evaluated what can be expected from incorporating the information from the DCISionRT test into standard clinical practice. Clinicians also ranked the top three factors influencing their recommendation. Clinician recommendation could be provided by the surgeon, radiation oncologist, or tumor board. Each patient had a recommendation from their treating physician but it was required that both the first and second recommendations were made by the same specialty. In this analysis, all samples were sent after primary surgery, with results disclosed to the ordering physician within 2–3 days of receipt of the samples by PreludeDx. Results were discussed with the patient by the treating physician and post-test clinical recommendations and patient preference for adjuvant RT were subsequently recorded. Clinicians also provided the ranked factors influencing their recommendation after DCISionRT testing. Overall, 697 patients were screened (48 screening failures, 22 withdrawals) and 627 were tested after BCS with complete data available. Eighty-eight cases were removed when case report forms (CRFs) for decision making were not completed by the same physician/specialty before and after testing, resulting in 539 patients who underwent post-surgery testing being evaluable for analysis.

The primary endpoint of the study was to identify the absolute percentage of all women for whom DCISionRT led to a change in physician treatment recommendations regarding adjuvant RT. Secondary endpoints included identifying key drivers of treatment recommendations with respect to RT based on clinicopathologic features, DCISionRT score, patient preference, and clinician specialty, and also determining the percentage of patients whose DCISionRT test led to a difference in physician treatment recommendations when stratified by clinical/pathologic factors (age, grade, tumor size, and margin status).

The percentage change in physician treatment recommendations was calculated, and McNemar’s test for paired data was used to assess the change in physician RT recommendations pre-test versus post-test. The percentage of patients recommended RT pre the DCISionRT test and the percentage of patients recommended RT post the DCISionRT test was calculated. Recommendation change was calculated as those patients who were initially recommended RT pre-test and then had a recommendation to omit RT post-test, and those patients initially recommended to omit RT pre-test and then had a recommendation to receive RT post-test. The percentage of patients who were initially recommended radiation therapy (RT) pre-test and were then recommended to omit RT post-test was calculated. Likewise, the percentage of patients who were initially recommended to omit RT pre-test and were then recommended to receive RT post-test was calculated. The impact of the DCISionRT results on patients meeting criteria such as the Radiation Therapy Oncology Group (RTOG) 9804 criteria (grade 1 or 2, size ≤ 2.5 cm, no close or positive margins, screen detected) or Eastern Cooperative Oncology Group (ECOG) E5194 criteria (low-risk: grade 1 or 2, size ≤ 2.5 cm, no close/positive margins; high risk: grade 3, size ≤ 1 cm, no close/positive margins) was evaluated.

Multivariate logistic regression analyses were used to assess the odds ratios (ORs) of factors leading to the pre-test and post-test RT recommendation. Pre-test covariates included age (≥ 70 years as well as < 50 years), grade (grade 3 vs. 1 or 2), tumor size (> 1 to ≤ 2.5 and > 2.5 vs. ≤ 1 cm), ethnicity (Hispanic vs. non-Hispanic), race (African American vs. Caucasian or other), family history (yes vs. no), necrosis (presence vs. absence or unknown), palpability (yes vs. no), hormone receptor (HR) status (negative and unknown vs. positive), margin status (close/positive vs. negative), and patient preference, as well as clinician specialty (surgeon/independent vs. radiation oncologist). Post-test covariates also included DS (elevated vs. low). Demographic and clinical characteristics of all patients were summarized. All analyses were performed in R Core Team.^13^

## Results

### Patient Characteristics

Table 1 presents patient and tumor characteristics. Patient age ranged from 30 to 87 years old, with a median age of 63 years old (first to third quartile, 54–70 years old). Median tumor size was 0.8 cm (first to third quartile, 0.4–1.4 cm). Overall, 32% of patients had grade 3 disease, 31% had a tumor larger than 1 cm, 17% had close or positive margins, and 5% of patients were HR-negative.

### Change in Radiation Therapy Recommendation

Table 2 presents the impact of DCISionRT testing on RT recommendations, by percentage, for the indicated patient subsets. Sixty-nine percent of all women (*n* = 539) were initially recommended RT pre-test (*n* = 374), and 46% of these women were recommended to not receive RT post-test (*n* = 165). Of those initially recommended to not receive RT (*n* = 165), 35% were recommended to receive RT post-test (*n* = 58). Consequently, there was a change in the RT recommendation for 42% of women (*n* = 539, *p* < 0.001) and a net reduction in the RT recommendation by 20%. A similar overall change (add or omit RT) in the RT recommendation post-DCISionRT testing was seen for radiation oncologists alone (44%), surgeons alone (49%), and radiation oncologists alone or in conjunction with tumor boards (38%). Independently, radiation oncologists were more likely to change a recommendation from ‘no RT’ to ‘yes RT’ (44%) compared with surgeons (28%), while surgeons were more likely to change their recommendation from ‘yes RT’ to ‘no RT’ (57%) compared with radiation oncologists (44%) after DCISionRT testing.

**Table 2:** Impact of DCISionRT on radiation therapy recommended

Recommending physician	*n*	RT recommended			Pre- to post-test change in RT recommended		Total change in RT recommended		
		Pre-test (%)	Post-test (%)	Net change (%)	Yes to no (%)	No to yes (%)	Overall change (%)	95% CI	*p*-Value
All	539	69	49	− 20	46	35	42	38–47%	< 0.001
Radiation oncologists (independently)	191	73	53	− 20	44	44	44	37–47%	< 0.001
All radiation oncologists (independently or with Tumor Board)	306	67	56	− 11	37	40	38	32–47%	0.001
Surgeons (independently)	232	72	39	− 33	57	28	49	42–47%	< 0.001

The directional change in RT recommendation is provided as the pre- to post-test change: (1) yes to no—the relative percentage of patients not recommended RT after DCISionRT testing of those who were initially recommended RT; and (2) no to yes—the relative percentage of patients who were recommended RT after DCISionRT testing of those who were initially not recommended RT

*RT* radiation therapy, *CI* confidence interval

Table 3 presents the impact of DCISionRT testing on adjuvant RT recommendations within common clinicopathologic features used by clinicians to decide on adjuvant RT. The overall change in RT recommendations was similar between different age groups, varying from 37% of women who were under 50 years of age (*n* = 82, *p* < 0.001) to 43% for women who were 50 years and older (*n* = 457, *p* < 0.001). With increasing age, the percentage of patients with RT recommended pre-test decreased from 79% to 52%, and among the corresponding cases initially not recommended RT, the percentage with RT recommended post-test increased from 12% to 53%. In patients < 50 years of age (*n* = 82), 79% (*n* = 65) were initially recommended RT pre-test; post-test, 43% (*n* = 28) of these cases were not recommended RT. Overall, in patients < 50 years of age, there was a corresponding net decrease of 31% in RT recommendations, from 65 patients pre-test to 39 patients post-test. For patients of 50 years of age and older, 67% were recommended RT pre-test (*n* = 306) and 33% were not (*n* = 151). Of patients initially not recommended RT (*n* = 151), 38% were recommended RT post-test (*n* = 57), whereas of patients recommended RT pre-test (*n* = 306), 46% were not recommended RT post-test (*n* = 141). Consequently, there was a 43% overall change in the RT recommendations (*n* = 457, *p* < 0.001) and an 18% (*n* = 82) net decrease in the RT recommendations. For patients 70 years of age and older (*n* = 138), there was a 10% net increase in the RT recommendations, from 72 patients pre-test to 86 patients post-test.

**Table 3:** Impact of DCISionRT on adjuvant radiation recommended by clinicopathologic features

Clinical factor	*n*	RT recommended			Pre- to post-test change in RT recommended		Total change in RT recommended		
		Pre-test (%)	Post-test (%)	Net change (%)	Yes to no (%)	No to yes (%)	Overall change (%)	95% CI	*p*-Value
*Age, years*									
< 50	82	79	48	− 31	43	12	37	27–47%	< 0.001
≥ 50	457	67	49	− 18	46	38	43	39–48%	< 0.001
≥ 60	324	64	53	− 11	42	43	42	37–48%	0.003
≥ 70	138	52	62	10	29	53	41	33–49%	0.06
*Grade*									
1 or 2	371	62	46	− 16	49	37	45	40–50%	< 0.001
3	168	86	55	− 31	39	22	37	30–44%	< 0.001
*Tumor size, cm*									
≤ 1	359	64	42	− 22	53	33	46	41–51%	< 0.001
≤ 2.5	487	67	47	− 20	47	36	43	39–48%	< 0.001
> 2.5	52	90	62	− 28	34	20	33	22–46%	< 0.001
*Margin status*									
Close (< 2 mm)	83	86	55	− 31	44	50	45	34–55%	< 0.001
Negative (≥ 2 mm)	447	66	46	− 20	47	34	43	38–47%	< 0.001

The directional change in RT recommendation is provided as the pre- to post-test change: (1) yes to no—the relative percentage of patients not recommended RT after DCISionRT testing of those who were initially recommended RT; and (2) no to yes—the relative percentage of patients who were recommended RT after DCISionRT testing of those who were initially not recommended RT

*RT* radiation therapy, *CI* confidence interval

With respect to grade, the RT recommendation was changed overall in 45% of patients with grade 1 or 2 disease (*n* = 371, *p* < 0.001) and in 37% of patients with grade 3 disease (*n* = 168, *p* < 0.001). For patients with grade 1 or 2 disease, there was a 16% net decrease (*n* = 229 pre-test vs. *n* = 169 post-test) in recommending RT, and for patients with grade 3 disease, there was a 31% net decrease (*n* = 145 pre-test vs. *n* = 93 post-test) in recommending RT. RT recommendations were changed overall in 46% (*n* = 359, *p* < 0.001) of patients with smaller tumors (≤ 1 cm) and in 33% (*n* = 52, *p* < 0.001) of patients with larger tumors (>2.5 cm). Clinicians initially recommended that 86% of patients with close margins should receive RT, compared with 66% of patients with clear margins (≥ 2 mm). A similar percentage of RT recommendations were changed pre- to post-test for women with close margins (45%, *n* = 83; *p* < 0.001) and clear margins (43%, *n* = 447; *p* < 0.001). Positive margins were not evaluated due to the limited number of cases (*n* = 9).

Table 4 presents the impact of DCISionRT for cohorts of patients based on traditional ‘low-risk’ features, by percentage, for indicated patient subsets. In patients meeting the criteria for RTOG 9804, 54% were recommended RT pre-test, with an overall change in RT recommended for 46% of patients (*n* = 268, *p* < 0.001) and a net decrease of 13% (*n* = 144 pre-test vs. *n* = 110 post-test) in recommending RT. Of the corresponding 46% of patients not recommended RT pre-test (*n* = 123), 36% were recommended RT post-test (*n* = 44). Similar findings were seen using the grade 1 or 2 ECOG E5194 study criteria. In contrast, of those patients meeting the grade 3 ECOG E5194 study criteria,^11^ 84% (*n* = 71) were recommended RT pre-test, with a 42% (*n* = 42) overall change in the RT recommendation (*n* = 85, *p* < 0.001) and a net 36% decrease in the RT recommendation (*n* = 71 pre-test vs. *n* = 45 post-test). Of those patients initially recommended RT (*n* = 71), 47% were not recommended RT post-test (*n* = 33). When looking at alternative criteria, patients with estrogen-positive disease who were older than 50 years of age had results similar to the grade 1 and 2 RTOG 9804 criteria findings.

**Table 4:** Impact of DCISionRT on treatment recommended by clinicopathologic features

Clinicopathologic criteria	*n*	RT recommended			Pre- to post-test change in RT recommended		Total change in RT recommended		
		Pre-test (%)	Post-test (%)	Net change (%)	Yes to no (%)	No to yes (%)	Overall change (%)	95% CI	*p*-Value
*RTOG 9804 criteria*									
Grade 1 or 2, size ≤2.5 cm, screen detected, no close or positive margins	268	54	41	− 13	54	36	46	40–52%	<0.001
*ECOG E5194 criteria*									
Grade 1 or 2, size ≤2.5 cm, no close or positive margins	280	54	41	− 13	53	35	45	39–51%	0.001
Grade 3, size ≤1 cm, no close or positive margins	85	84	48	− 36	47	21	42	32–53%	<0.001
*Alternative criteria*									
Grade 3, size ≤2.5 cm, screen detected, no close or positive margins	116	85	54	− 31	42	28	40	31–49%	<0.001
Age ≥50 years, size ≤2.5 cm, estrogen receptor-positive, no close or positive margins	231	58	46	− 12	51	41	47	40–53%	0.004

The directional change in RT recommendation is provided as the pre- to post-test change: (1) yes to no—the relative percentage of patients not recommended RT after DCISionRT testing of those who were initially recommended RT; and (2) no to yes—the relative percentage of patients who were recommended RT after DCISionRT testing of those who were initially not recommended RT

*RT* radiation therapy, *CI* confidence interval, *RTOG* Radiation Therapy Oncology Group, *ECOG* Eastern Cooperative Oncology Group

Table 5 presents changes in RT recommendations according to the DS Low and DS Elevated risk groups. Overall, patients with a DS Low risk score (63% of all patients) had a 45% net reduction (*n* = 248 pre-test vs. *n *= 95 post-test, of 341) in RT recommendations post-DCISionRT testing, while those with a DS Elevated risk score (37% of patients) had a 21% absolute increase (*n* = 126 pre-test vs. *n *= 167 post-test, of 198) in RT recommendations post-DCISionRT testing. When evaluating patients meeting the RTOG 9804 and ECOG E5194 criteria, similar changes were seen with the DS Low risk results compared with all patients. However, in DS Elevated risk patients, there were greater increases in RT recommendations in the RTOG 9804 and ECOG E5194 subpopulations compared with the entire population.

**Table 5:** Impact of DCISionRT on treatment recommended for the low-risk cohorts

Clinicopathologic criteria	*n*	Low risk (DS ≤3)						Elevated risk (DS >3)					
		RT recommended				Pre- to post-test change in RT Recommended		RT recommended				Pre- to post-test change in RT Recommended	
		*n*	Pre-test (%)	Post-test (%)	Net change (%)	Yes to no (%)	No to yes (%)	*n*	Pre-test (%)	Post-test (%)	Net change	Yes to no (%)	No to yes (%)
All patients	539	341	73	28	− 45	65	9	198	64	84	21	7	69
*RTOG 9804 criteria*													
Grade 1 or 2, size ≤2.5 cm, screen detected, no close or positive margins	268	172	59	20	− 39	72	9	96	44	78	34	12	70
*ECOG E5194 criteria*													
Grade 1 or 2, size ≤2.5 cm, no close or positive margins	280	182	60	21	− 39	70	8	98	44	79	35	12	71
Grade 3, size ≤1 cm, no close or positive margins	85	61	85	33	− 52	62	0	24	79	88	8	5	60
*Alternative criteria*													
Not RTOG 9804	271	169	86	36	− 51	60	9	102	82	90	8	5	67

The directional change in RT recommendation is provided as the pre- to post-test change: (1) yes to no—the relative percentage of patients not recommended RT after DCISionRT testing of those who were initially recommended RT; and (2) no to yes—the relative percentage of patients who were recommended RT after DCISionRT testing of those who were initially not recommended RT

*RT* radiation therapy, *RTOG* Radiation Therapy Oncology Group, *ECOG* Eastern Cooperative Oncology Group, *DS* decision score

Furthermore, when assessing the continuous DS result post-testing, 26% of patients were recommended RT when they had lower scores (DS ≤ 2), while 95% of patients were recommended RT when they had higher scores (DS ≥ 4).

### Logistic Regression of Factors Influencing Treatment Decisions

**Fig. 1:**
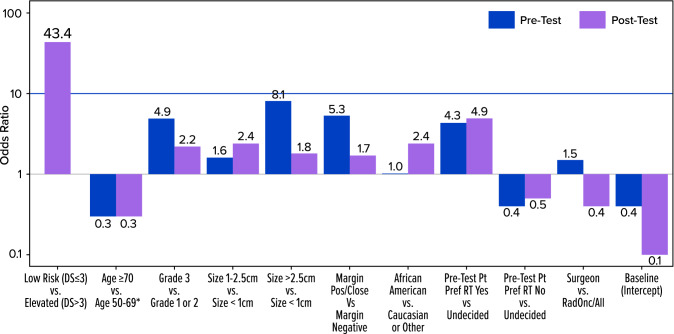
Factors associated with the recommendation of RT before and after DCISionRT. *RT* radiation therapy. See Table 6 for complete list of factors associated with decision making, including non-significant factors

Figure 1 and Table 6 present multivariable logistic regression analyses of factors associated with the recommendation of RT pre- and post-DCISionRT testing. Pre-testing factors associated with increased RT recommendations included grade 3 versus grade 1 or 2 (OR 4.9), size > 1 and ≤ 2.5 cm versus size ≤ 1 cm (OR 1.6), size >2.5 cm versus ≤ 1 cm (OR 8.1), margin status (close/positive) versus negative (OR 5.3), and patient preference to receive RT (OR 4.3). Pre-testing, the factors that decreased the likelihood of an RT recommendation were patient age ≥ 70 years (OR 0.3) and patient preference to not receive RT (OR 0.4). Post-DCISionRT testing, factors associated with an increased likelihood of an RT recommendation included size >1 cm versus ≤ 1 cm (OR 2.4), grade 3 versus grade 1 or 2 (OR 2.2), African American race (OR 2.4), initial patient preference to receive RT (OR 4.9), and DCISionRT DS Elevated risk (OR 43.4). Post-testing, factors associated with a decreased likelihood of an RT recommendation were patient aged ≥ 70 years (OR 0.3) and an initial patient preference to not receive RT (OR 0.5). Following DCISionRT testing, surgeons were less likely to recommend RT than radiation oncologists (OR 0.4). The DCISionRT score had a substantially greater impact (by eightfold) on RT recommendations (OR 43.4) in comparison with all other factors.

**Table 6:** Factors associated with recommendation for radiation therapy before and after DCISionRT

Factor	Pre-DCISionRT			Post-DCISionRT		
	OR^a^	95% CI	*p*-Value	OR^a^	95% CI	*p*-Value
DS > 3 (elevated) vs. DS ≤ 3 (low)				43.4	21.6–94.1	< 0.001
*Age, years*						
≥ 70 vs. ≥ 50 and < 70	0.3	0.2–0.5	< 0.001	0.3	0.2–0.6	0.003
< 50 vs. ≥ 50 and < 70	1.3	0.7–2.8	0.41	1.4	0.7–2.8	0.31
*Grade*						
3 vs. 1 or 2	4.9	2.8–9.5	< 0.001	2.2	1.3–3.8	0.006
*Necrosis*						
Present vs. absent or unknown	1	0.7–1.7	0.92	1	0.6–1.7	0.94
*Estrogen receptor*						
ER-negative vs. ER-positive	2.2	0.7–1.7	0.48	0.53	0.4–5.4	0.53
Unknown vs. ER-positive	1	0.6–1.7	0.96	1.2	0.7–1.9	0.58
*Size, cm*						
>1 to ≤2.5 vs. ≤1	1.6	1.0–3.0	0.07	2.4	1.4–4.2	0.002
>2.5 vs. ≤1	8.1	2.8–29.7	< 0.001	1.8	0.8–4.5	0.15
*Margin status*						
Close or positive vs. negative	5.3	2.8–12.4	< 0.001	1.7	0.9–3.3	0.1
*Palpable*						
Yes. vs. no	3.1	0.6–18.9	0.18	0.9	0.3–3.0	0.91
*Family history of BC*						
Yes vs. no	1.2	0.7–1.8	0.52	1.2	0.7–1.8	0.55
*Ethnicity*						
Hispanic vs, non-Hispanic	1.1	0.2–5.8	0.95	1.6	0.3–10.1	0.58
*Race*						
African American vs. Caucasian or other	1	0.5–1.8	0.98	2.4	1.0–4.5	0.02
*Comorbidity*						
Present vs. absent	0.3	0.1–1.1	0.07	1.1	0.2–4.4	0.9
*Patient preference*						
Pre-Test RT: yes vs. undecided	4.3	1.5–15.8	0.01	4.9	1.9–12.8	0.001
Pre-Test RT: no vs. undecided	0.4	0.3–0.8	< 0.001	0.5	0.3–0.8	0.01
*Physician type*						
Surgeon, independent vs. radiation oncologist, all	1.5	1.02–2.5	0.11	0.4	0.2–0.6	< 0.001
*Endocrine therapy*						
Recommended vs. not recommended	1	0.7–2.5	0.94	1	0.5–1.9	0.97

*OR* odds ratio, *CI* confidence interval, *DS* decision score, *ER* estrogen receptor, *RT* radiation therapy

^a^Odds ratio from logistic regression analysis

## Discussion

The results of this analysis demonstrate several key findings. First, utilization of the DCISionRT test led to a significant overall change in the RT recommendations, with a substantial absolute net decrease in RT recommended. However, results changed RT recommendations for both patients who were initially recommended to omit and who were recommended to receive RT pre-test across all specialties. A significant change in RT recommendations post-test was seen across all age groups, tumor grades, and tumor sizes, as well as within patients meeting traditional ‘low-risk’ clinicopathologic criteria used in randomized and prospective studies.^6^–^8^ Post-DCISionRT testing, elevated versus low DCISionRT score (DS>3 vs. DS ≤ 3) was the strongest factor for an RT recommendation (OR 43.1) compared with all other factors (age, grade, tumor size, margin status), as well as patient preference.

The utility of the DCISionRT test result on RT recommendations is reflected by the percentage of patients recommended RT as a function of the DCISionRT score. From pre-test to post-test within the DS Low (DS ≤ 3) and DS Elevated (DS > 3) risk groups, there were significant absolute overall and net changes in treatment recommendations. The pre-testing RT recommendations were moderately high in both the DS Low and DS Elevated risk groups (73% and 64%, respectively). The RT recommendation was reduced in the DS Low risk group (absolute 45% reduction, to 28% post-test). However, it is important to also note that the RT recommendation was increased for patients in the DS Elevated risk group (DS > 3), by an absolute 21–35% post-test. Furthermore, for DS results > 4, clinicians recommended that 95% of patients receive RT. This study demonstrates that the DCISionRT test substantially changed RT treatment recommendations for 539 patients treated at 44 study sites.

### Study Implications

It is important to frame the changes in RT recommendations within the PREDICT study with the goal of DCIS treatment and the clinical outcomes of the DCISionRT test. The goal of therapy for DCIS is to prevent the development of invasive breast cancer events. DCIS of the breast represents a broad biologic spectrum of disease and the best form of local therapy is still debated today. While the majority of patients do not develop invasive cancer, it has historically been difficult to predict which women will progress and which women will not. The uncertainty associated with subsequent breast cancer risk has historically complicated DCIS treatment decisions for both patients and clinicians, leading to the broad utilization of RT following BCS.

The DCISionRT test assesses the risk of subsequent progression to invasive breast cancer events as well as DCIS events and has been validated in women treated with BCS, with or without RT. Weinmann et al. evaluated the test in 455 patients diagnosed with DCIS and treated with BCS, with and without RT.^11^ The study determined that the DCISionRT result was associated with 10-year total and invasive breast event risk after adjusting for RT. The test addressed the need for assessing subsequent invasive breast cancer event risk, where increasing DS (hazard ratio [HR] 4.0 invasive) was associated with increasing 10-year invasive breast cancer events, consistent with the initial publication (HR 4.2 invasive).^11^,^12^ Patients with DS Elevated risk were associated with increased risk for invasive breast cancer and total recurrences compared with low-risk patients. Importantly, DCISionRT did not simply correlate with traditional clinicopathologic features or ‘low-risk criteria’ used to assess risk; instead 40–50% of patients with low-risk factors were found to have elevated DS risk scores.^11^,^12^ In particular, the present analysis showed the impact of DCISionRT testing on patients with grade 3 DCIS, who are commonly recommended to receive RT based on the ECOG E5194 trial, which demonstrated a long-term local recurrence risk of close to 24%; however, the present analysis demonstrates that some of these patients may not require RT.

Previously, alternative multigene assays (example Oncotype DCIS) have been evaluated. Solin et al. presented outcomes from ECOG E5194 demonstrating the ability of such assays to predict the risk of ipsilateral breast events and invasive breast events; however, the data were unable to demonstrate a differentiation of benefit or lack thereof from adjuvant RT.^14^ Unfortunately, subsequent studies demonstrated that this assay did not provide discrimination of risk of ipsilateral events compared with traditional clinical and pathologic features utilized in a nomogram.^15^ In contrast, a recent analysis of DCISionRT evaluated patients meeting RTOG 9804 and ECOG E5194 criteria and found that the 10-year risk of invasive cancer recurrences was only impacted by 1.1–1.5% with the addition of RT for DS Low risk patients, but was dramatically impacted for DS Elevated risk patients by 8.3–14.7%.^16^ Similarly, a previous study found that in patients in the DS Elevated risk group, those not receiving RT had a 21% risk of invasive breast cancer events at 10 years, while those receiving RT had a 6% risk. However, for patients in the DS Low risk group, 10-year risk of invasive breast cancer events was 5% for patients treated without RT and 3% for patients treated with RT.^11^ In aggregate, the DCISionRT test has been validated in over 1400 patients from four study cohorts.^11^,^12^,^17^ Consistent with early breast cancer trials, the average RT risk reduction in these cohorts was 50% pre-test; however, those patients with elevated test scores (DS >3) had clinically elevated risks and a 70% relative risk reduction with RT. This corresponded to an absolute risk reduction of 9–15% in 10-year invasive breast cancer event rates. Those with lower test scores (DS ≤ 3) had low clinical risks and minimal differences in 10-year invasive cancer event rates for patients treated with versus without RT (1–2%).

Typically, clinicians have based their RT recommendations on clinicopathologic features, consistent with the factors driving RT recommendations pre-test. The reported differentiation in 10-year risks provided by DCISionRT, depending on treatment, is a tool that can provide clinicians with relevant data to guide clinical management of DCIS treatment, and explains why physicians across all disciplines utilized the DCISionRT result to change their clinical recommendation regarding the role of RT. However, these findings also support that decision making with respect to adjuvant RT requires moving beyond clinicopathologic features alone.

When evaluating patients commonly defined as low-risk, using criteria such as the RTOG 9804 and ECOG E5194, our results demonstrated significant changes (42–46%) in RT recommendations with DCISionRT testing. Additionally, when segregating patients by DS risk within patients meeting the low-risk clinicopathologic criteria, substantial changes were noted, both with respect to reducing the rates of RT recommendation for patients with DS Low risk scores and increasing the rates of RT recommendations for patients with DS Elevated risk scores. These findings support that DCISionRT adds critical information beyond traditional clinicopathologic features used when deciding to recommend or omit RT, which has been validated with long-term outcomes noted above.^16^

Moving forward, one question pertains to patients who are at persistently elevated risk of recurrence following standard treatment for DCIS. In the DS Elevated risk group, the test stratified 10-year invasive recurrence risks from 7 to 40%.^11^,^12^,^17^ A recent study including three cohorts of patients treated from 1986 to 2008 identified a subset of patients with a biological good or poor response subtype with a higher local recurrence risk after standard therapy.^17^ Patients with DS Elevated risk and a biological good response subtype defined by a panel of biomarkers had excellent outcome compared with patients undergoing BCS without RT (22% 10-year total risk of recurrence) or patients with a poor response subtype receiving standard therapy. Patients with a good response subtype treated with BCS and RT had a 5% 10-year total risk of recurrence. Those patients with DS Elevated risk and a biological poor response subtype had higher rates of recurrence after BCS with RT (23% 10-year total risk of recurrence), suggesting that there may be a subset of DCIS patients for whom additional treatment strategies may be needed.^17^

### Study Limitations

There are limitations to this analysis. The primary objective was to evaluate the impact of testing on treatment decision recommendations; however, long-term clinical outcomes, including clinical outcomes as well subsequent resource utilization associated with the treatment decisions, are currently unavailable from the registry but are planned for assessment when longer follow-up is available. Additionally, data on RT recommendations were only based on two time points, the first being post-surgery/pre-testing and the second being post-surgery/post-testing. Finally, patient- and physician-reported outcomes regarding quality and satisfaction with decision making are pending at this time.

## Conclusions

Utilization of DCISionRT demonstrated a substantial overall change (42%) in the recommendation for RT for DCIS patients undergoing BCS, resulting in a 20% absolute net decrease in RT recommendation. In comparison with traditional clinicopathologic features, the DCISionRT score was strongly associated with RT therapy recommendation. Importantly, post-test RT was recommended to 35% of women not recommended to receive radiation pre-test. The results demonstrated that physicians incorporated DCISionRT testing into routine practice for DCIS treatment management and viewed the DCISionRT score as the most impactful factor for making adjuvant RT recommendations, with other factors of importance being patient preference, size, and grade.

## Supplementary Information

Below is the link to the electronic supplementary material.

Supplementary file1 (DOCX 15 kb)
