# Supplementary material for: The Clinical Utility of DCISionRT® on Radiation Therapy Decision Making in Patients with Ductal Carcinoma In Situ Following Breast-Conserving Surgery
Source: Ann Surg Oncol. 2021 Apr 5;28(11):5974–84. doi: 10.1245/s10434-021-09903-1 (PMC8526470; doi:10.1245/s10434-021-09903-1)
Supplement: Supplementary file 1 — Supplementary file1 (DOCX 15 kb) [file 10434_2021_9903_MOESM1_ESM.docx]

**Supplemental Table 1:** Enrolling Institutions at the Time of This Analysis

| **Institution** | **Location** |
| --- | --- |
| Arizona Center for Cancer Care | Peoria, AZ |
| Ironwood Cancer and Research Centers | Scottsdale, AZ |
| Good Samaritan Hospital | Los Gatos, CA |
| Memorial Medical Center Sutter Health | Modesto, CA |
| Hoag Memorial Hospital Presbyterian | Newport Beach, CA |
| John Wayne Cancer Institute | Santa Monica, CA |
| University of Colorado Denver | Aurora, CO |
| Shaw Cancer Center | Edwards, CO |
| AdventHealth Hematology & Oncology | Altamonte Springs, FL |
| Regional Breast Care | Fort Myers, FL |
| St. Joseph's Hospital Tampa | Tampa, FL |
| University of South Florida | Tampa, FL |
| WellStar Health System | Marietta, GA |
| Memorial Health University Medical Center | Savannah, GA |
| Oncology Associates at Mercy Medical Center | Cedar Rapids, IA |
| UnityPoint Health | Cedar Rapids, IA |
| Alexian Brothers Medical Center (AMITA Health) | Elk Grove Village, IL |
| Advocate Health Care | Park Ridge, IL |
| Northwestern Medicine Central DuPage Hospital | Winfield, IL |
| Dana-Farber Cancer Institute | Boston, MA |
| DFCI South Shore Hospital | South Weymouth, MA |
| MedStar Health Research Institute | Baltimore, MD |
| Mercy Medical Center | Baltimore, MD |
| Beaumont Health Royal Oak | Royal Oak, MI |
| Comprehensive Breast Care | Troy, MI |
| MD Anderson Cancer Center at Cooper | Camden, NJ |
| Atlantic Health Care Morristown Medical Center | Morristown, NJ |
| Maimonides Cancer Center | Brooklyn, NY |
| Cleveland Clinic Akron General | Akron, OH |
| Summa Health | Akron, OH |
| St Luke's University Health Network Bethlehem | Bethlehem, PA |
| Bon Secours St Francis Cancer Center | Greenville, SC |
| The West Clinic | Germantown, TN |
| Knoxville Comprehensive Breast Center | Knoxville, TN |
| Nashville Breast Center | Nashville, TN |
| Dallas Surgical Group | Dallas, TX |
| Baylor College of Medicine | Houston, TX |
| Elizabeth Bonefas, MD | Houston, TX |
| Houston Methodist Hospital | Houston, TX |
| Southwest Surgical Associates | Houston, TX |
| Inova Schar Cancer Institute | Fairfax, VA |
| Bon Secours Virginia Breast Center | Midlothian, VA |
| Summit Cancer Centers | Spokane Valley, WA |
| Aspirus Regional Cancer Center | Wausau, WI |

Participating institutions ordered by the state in which the institutions reside.
